# Inflammatory dilated cardiomyopathy associated with psoriasis: a case report

**DOI:** 10.1186/s13256-023-04207-2

**Published:** 2023-11-13

**Authors:** Hamidreza Riasi, Emad Asgari Jafarabadi, Hadis Enayati, Ali Fanoodi, Shiva Salehi, Ali-Reza Jamshidi, Forod Salehi, Azam Rezaee

**Affiliations:** 1 Department of Neurology, School of Medicine, Cardiovascular Diseases Research Center, Birjand University of Medical Sciences, Birjand, Iran; 2 Department of Pediatrics, School of Medicine, Birjand University of Medical Sciences, Birjand, Iran; 3 Student Research Committee, School of Medicine, Birjand University of Medical Sciences, Birjand, Iran; 4 Medical Student, College of Medicine, Baghyatallah University of Medical Sciences, Tehran, Iran; 5 Department of Pediatrics, School of Medicine, Cardiovascular Diseases Research Center, Birjand University of Medical Sciences, Birjand, Iran; 6 Assistant Professor of Rheumatology, Department of Internal Medicine, School of Medicine, Birjand University of Medical Sciences, Birjand, Iran; 7 Clinical Research Development Unit, Vali-E-Asr Hospital, Birjand University of Medical Sciences, Birjand, Iran; 8 Medical School of Birjand University of Medical Sciences, Ghafari Blvd, Birjand, South Khorasan Iran

**Keywords:** Psoriasis, Inflammatory cardiomyopathy, Heart failure, Case report

## Abstract

**Background:**

Psoriasis is a chronic inflammatory skin disease with a genetic basis. Psoriasis is accepted as a systemic, immune-mediated disease. Hypertension, obesity, metabolic disorders including diabetes mellitus and hyperlipidemia, and psychiatric disorders are more prevalent among children with psoriasis compared to children without psoriasis. In this study, we report a case of dramatic response of inflammatory cardiomyopathy to anti-inflammatory treatment of psoriasis; which might reveal similar pathogenesis basis of these two diseases.

**Case presentation:**

A 9-year-old Caucasian boy presenting with signs and symptoms of heart failure refractory to conventional therapies was admitted to our pediatric cardiology service. As the patient also had psoriasis, and considering the fact that there might be an association between the two conditions, immunosuppressive drugs were administered, which led to a dramatic improvement in heart function.

**Conclusions:**

The results of this study add to evidence linking psoriasis with inflammatory dilated cardiomyopathy. Clinicians, particularly cardiologists, must pay special attention to the cardiac complications of systemic diseases.

## Introduction

Psoriasis is a chronic inflammatory skin disease with a genetic basis [1]. Several clinical subtypes of psoriasis have been described; among them, chronic plaque (psoriasis vulgaris) is the most common type, occurring in about 90% of affected patients. Areas of the body most commonly affected are the back of the forearms, shins, navel area, and scalp [2, 3]. Most affected children have mild to moderate disease, so topical therapy successfully controls the disease [4].

Despite lack of enough epidemiologic data about psoriasis, it is considered to be a relatively common childhood dermatologic disease. The worldwide prevalence of psoriasis is 2–3% [5]; this value is estimated to be 1.3–2.5% among Iranians [6]. Data suggest that in 30–32% of patients, symptoms start before 15 years. Meanwhile, different studies have reported different data regarding the age of onset. Psoriasis has a genetic background and a considerable number of patients with psoriasis have a positive family history [4, 7].

Recent evidence suggests that psoriasis is accepted as a systemic, immune-mediated disease associated with an increased prevalence of various cardiovascular comorbidities. Hypertension, obesity, metabolic disorders including diabetes mellitus and hyperlipidemia, and psychiatric disorders are more prevalent among children with psoriasis compared to children without psoriasis [8]. Psoriasis is also a risk factor for increased morbidity and mortality from cardiovascular disease [9].

We present a rare case of dilated cardiomyopathy associated with psoriasis in a 9-year-old child with dramatic response to anti-inflammatory treatment of psoriasis which suggests similar pathogenesis between these two diseases.

This case report was approved by the Institution’s Research Ethics Committee (Approval ID: IR.BUMS.REC.1399.356), and has been based on the CARE reporting guidelines [10]. Moreover, written informed consent was obtained from the patient's legal guardian.

### Case presentation

**Fig. 1:**
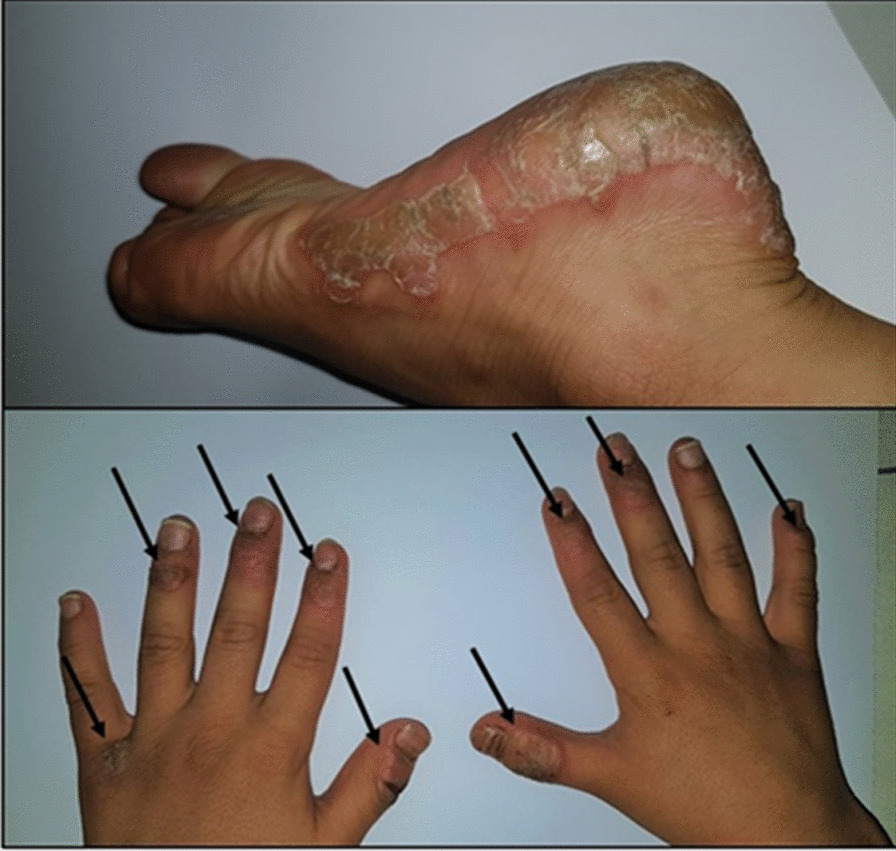
Psoriatic lesions in foot and hands (the arrows). In this patient, psoriatic lesions were found since the age of two, which had a chronic course and no flare-ups were reported. The psoriatic lesions have been partially controlled through the application of emollients and topical corticosteroids

A 9-year-old Caucasian boy with past medical history of plaque psoriasis since the age of two, on topical steroids and emollients, presented to an outpatient clinic with fatigue, abdominal pain, and intractable nausea for 2 weeks. The abdominal pain was constant, without any radiation, aggravated by physical activity, and mostly presented in the right upper quadrant. No significant family or psychosocial history was found, including family history of psoriasis or familial cardiomyopathy. The weight of the patient was 27 kg (25–50th centile), and the vital signs were stable (no tachycardia, tachypnea, or hypotension were found). Epigastric and right upper quadrant tenderness, hepatomegaly, and muffled heart sounds were evident during physical examination. Psoriatic lesions of hands and feet were noticed during detailed physical examination (Fig. 1). The psoriatic lesions have been partially controlled through the application of emollients and topical corticosteroids. In this patient, psoriatic lesions were found since the age of two, which had a chronic course and no flare-ups were reported.

**Fig. 2:**
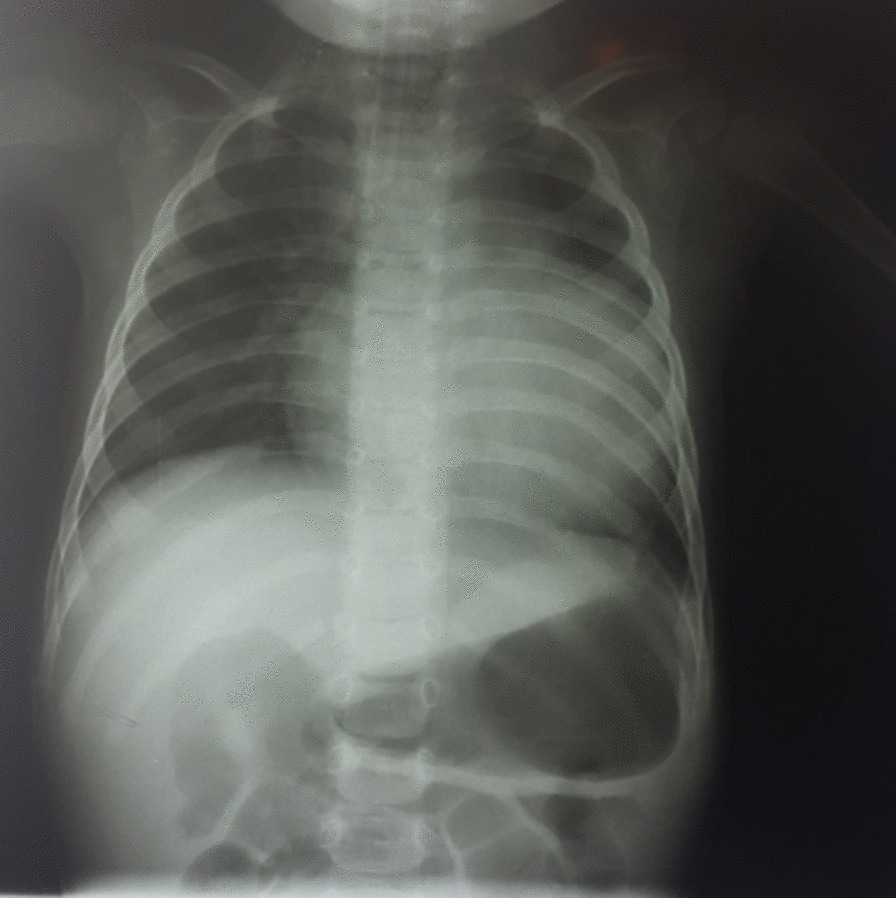
Chest X-ray PA view showing cardiomegaly and hepatomegaly in the patient

**Fig. 3:**
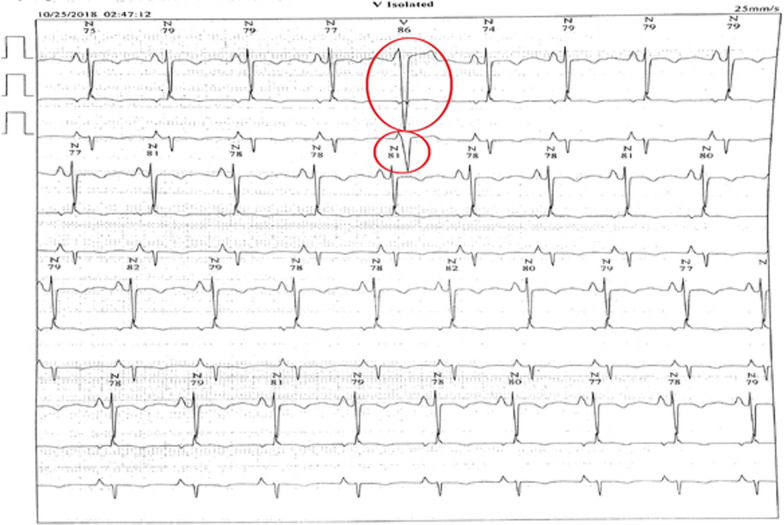
Holter monitoring showing premature ventricular contractions (PVCs) in D2 lead in the patient (the red circles)

**Fig. 4:**
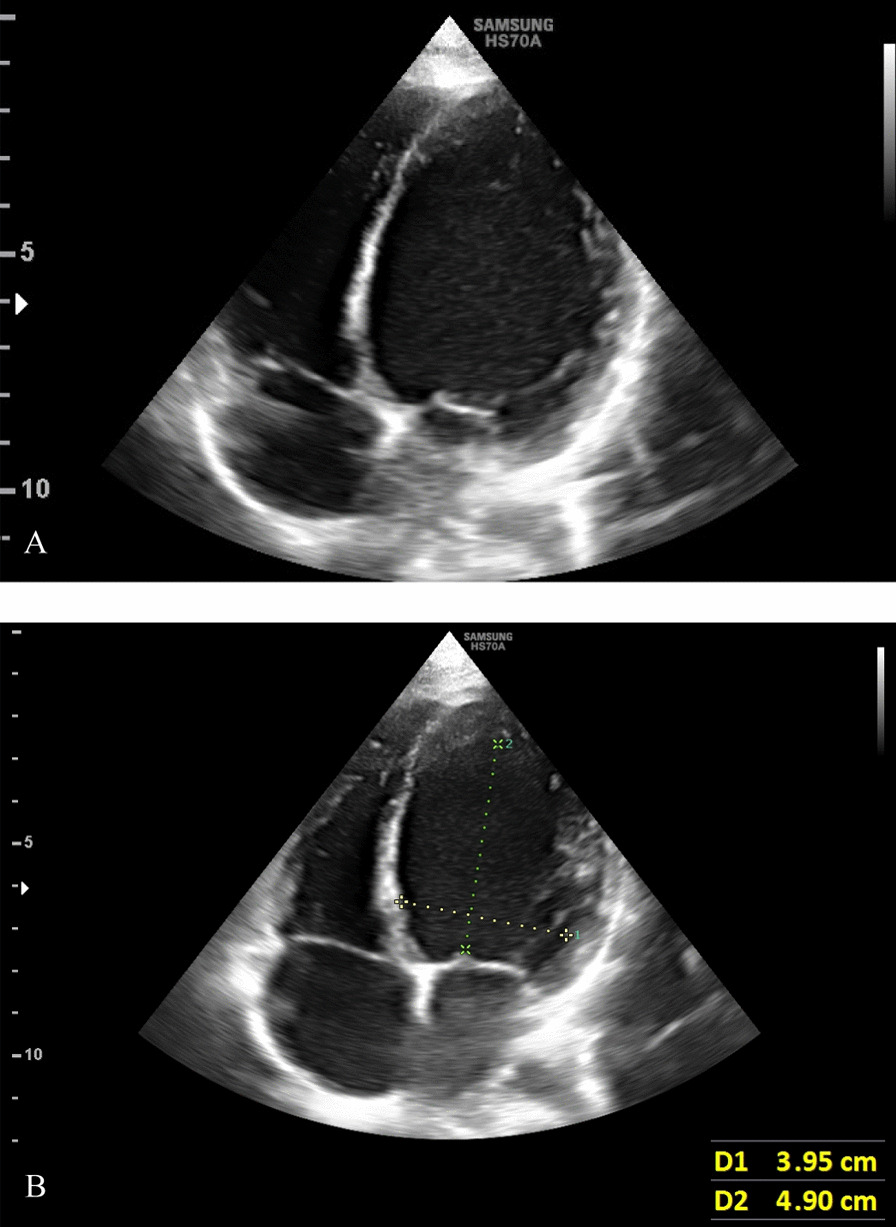
Echocardiography of the patient. **A** Left ventricle dilatation and its large size compared with the right ventricle and other cavities are evident. Ejection Fraction (EF) of 39%, and severe mitral regurgitation were also reported. **B** The left ventricular sphericity index (LVSI) is 0.80 (4.90 cm/3.95 cm). LVSI is measured by the LV short-to-long-axis dimension ratio in end-diastolic apical four-chamber view. LVSI has been validated as a direct measure of LV remodeling in patients with dilated cardiomyopathy [28]

**Fig. 5:**
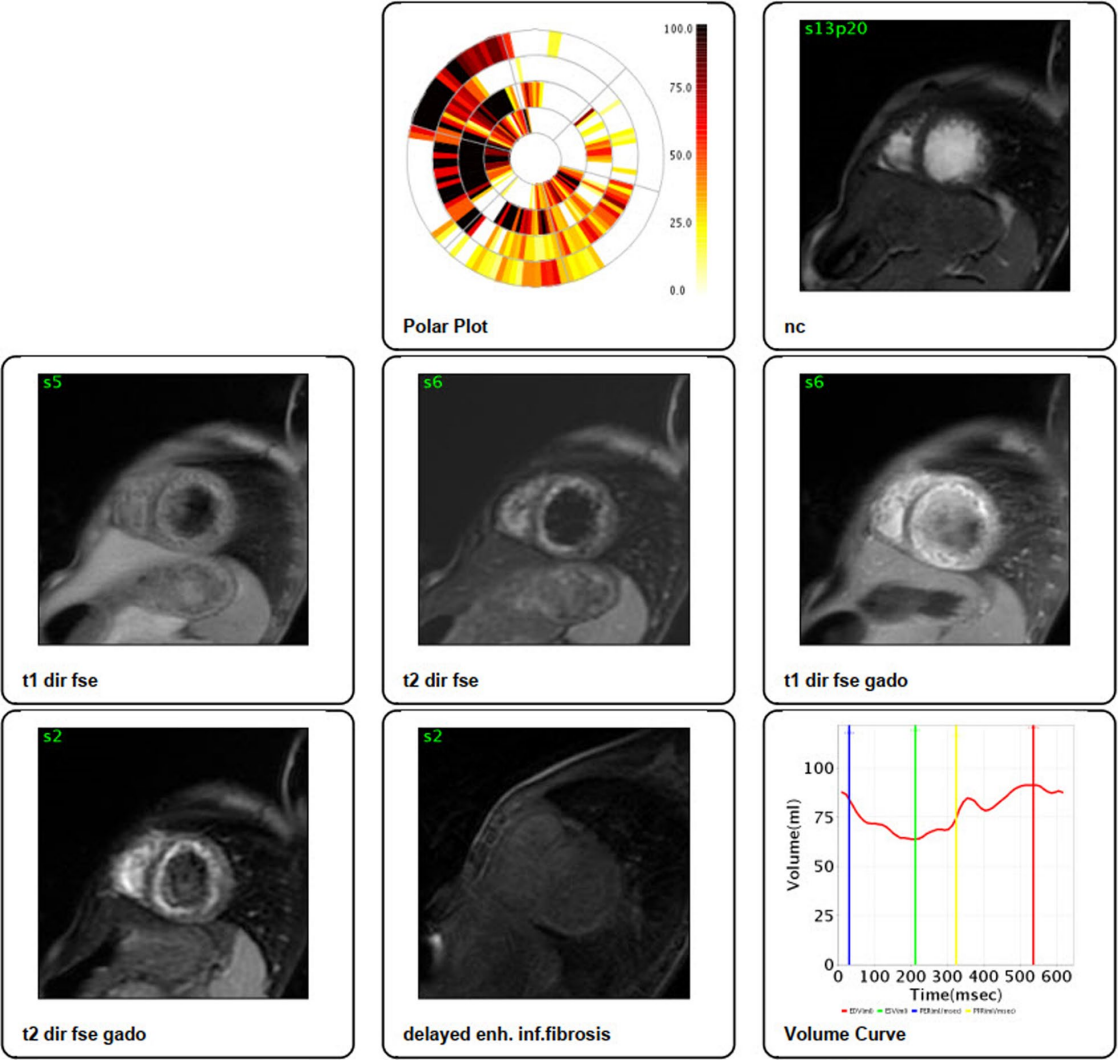
Cardiac Magnetic Resonance Imaging (MRI) and Steady-State Free Precession (SSFP) showed cardiac chamber dilation. Left ventricular thickness was mildly thinned associated with increased end-diastolic volume in right ventricle. In addition, low signal intensity was seen in T1, however, high signal intensity and decreased EF was seen in T2. Late Gadolinium Enhancement (LGE) showed linear mid-myocardial enhancement in interventricular septum (LV End-Systolic Volume = 103.1 ml; LV End-Diastolic Volume = 137.2 ml; LV Ejection Fraction = 25%; Stroke Volume = 34.1 ml; Cardiac Output = 3.4 l/min; Cardiac Index = 3.9 l/min/m^2^)

He was seen in outpatient pediatric office and was admitted to the hospital for further work-up. Extensive laboratory studies were performed; liver enzymes, serum cholesterol level, troponin, and other cardiac enzymes were elevated. The results of laboratory evaluations are presented in Table 1. Ultrasonography scan detected free fluid in the right paracolic gutter and Morison`s pouch, and right mild pulmonary effusion was also present. A chest radiograph was obtained, in which cardiomegaly was noted (Fig. 2). Therefore, A pediatric cardiologist was consulted. Occasional premature ventricular contractions (PVC) were evident in standard electrocardiography. According to the consultation with pediatric cardiologist, exercise tolerance test and 24-hours Holter monitoring were recommended; recurrent PVCs were detected in both studies (Fig. 3). Echocardiography revealed dilated cardiomyopathy with an Ejection Fraction (EF) of 39%, and also severe mitral regurgitation (Fig. 4). In order to further assess the cause of heart failure, cardiac Magnetic Resonance Imaging (MRI) was performed; which confirmed dilated cardiomyopathy as the underlying pathologic condition (Fig. 5). As endomyocardial biopsy is not part of routine practice, it was not performed.

**Table 1:** Blood examination findings of the patient

Test	Level	Test	Level	Pathogen	Result of Real-time PCR
pH	7.420	**SGOT**	**55 U/L**	Adenovirus	Undetectable
**pCO**_**2**_	**31.2 mmHg**	SGPT	38 U/L	CMV	Undetectable
**pO**_**2**_	**44.4 mmHg**	**CK-MB**	**64 IU/L**	EBV	Undetectable
**HCO**_**3**_	**20.3 mEq/L**	CPK	249 IU/L	HSV 1 & 2	Undetectable
**O**_**2**_** sat**	**80.8%**	PTT	28	VZV	Undetectable
BE	− 2.9 mEq/L	**TPI**	**Positive**	Enterovirus	Undetectable
Hgb	12.8 gr/dl	Lipase	11 U/L	Parechovirus	Undetectable
Hct	38.7%	**CRP**	**26 mg/L**	Human herpes virus 6 & 7	Undetectable
Alb	3.9 g/dL	ASO	Negative	Parvovirus B19	Undetectable
FANA	Negative	TSH	2.54 mU/L	Urine & plasma amino acid chromatography	Normal
NH_3_	52.5 µg /dL	FT4	1.6 ng/dL		
Total proteins	6.0 g/dL	**Cholesterol**	**320**		

*pH* Hydrogen ion concentration; *pCO*_*2*_ Partial pressure of carbon dioxide; *pO*_*2*_ Partial pressure of oxygen; *HCO*_*3*_ Bicarbonate; *O*_*2*_* sat* Oxygen saturation; *BE* Base excess; *Hgb* Hemoglobin; *Hct* Hematocrit; *Alb* Albumin; *FANA* Fluorescent antinuclear antibody; *NH*_*3*_ Ammonia; *SGOT* Serum glutamic-oxaloacetic transaminase; *SGPT* Serum glutamic-pyruvic transaminase; *CK-MB* Creatine kinase-MB; *CPK* Creatine phosphokinase; *PTT* Partial Thromboplastin Time; *TPI* Triose phosphate isomerase; *CRP* C-reactive protein; *ASO* Anti-streptolysin O; TSH Thyroid-stimulating hormone; *FT4* Free Thyroxine; *CMV* Cytomegalovirus; *EBV* Epstein–Barr virus; *HSV 1 & 2* Herpes Zoster virus 1 & 2; *VZV* Varicella-Zoster Virus

*The abnormal test results are bolded

Heart failure treatment with digoxin (10 μg/kg daily), furosemide (1–3 mg/kg BD), spironolactone (1–4 mg/kg BD), aspirin (3–5 mg/kg daily), carvedilol (1–2 mg/kg BD), atorvastatin (0.5–1 mg/kg daily), and losartan (1–3 mg/kg BD) was initiated, which resulted in a transient improvement in cardiac function, and the EF was improved to 45%. The initial improvement was transient and it was followed by the development of hepatomegaly, ascites, and lower limbs edema; the EF was reduced to 27%.

After two weeks of hospitalization, as psoriatic lesions of hands and feet were present, pediatric rheumatologist was consulted. Therefore, prednisolone (1–2 mg/kg BD) and azathioprine (2–2.5 mg/kg daily) were added to the therapeutic regimen. Treatment with anti-inflammatory drugs resulted in a remarkable improvement in heart failure, and EF was interestingly improved (65%). Following the improvement of patient`s condition, we were able to reduce the dose of heart failure medications. The discharge medication list included atorvastatin (0.5–1 mg/kg daily), digoxin (10 μg/kg daily), furosemide (1–3 mg/kg BD), spironolactone (1–4 mg/kg BD), and hydrochlorothiazide (1–3 mg/kg daily). In five-year follow-up visits, psoriatic lesions were still present. The patient used prednisolone (1–2 mg/kg BD) and azathioprine (2–2.5 mg/kg daily) during these years in order to control psoriatic lesions. However, no recurrence of heart failure or reduction of EF were found.

## Discussion

The left ventricle end-systolic and end-diastolic diameters of patients having psoriatic arteriopathy are statistically different from those of the healthy volunteers in the control group [11]; this fact highlights the effect of chronic inflammation on myocardial function. Endothelial dysfunction and atherosclerotic arteriopathy impair myocardial blood flow and leads to ischemia and impaired function, and the inflammatory cardiomyopathy that accompanies psoriasis could further deteriorate heart function. In a study performed by Cox *et al*. in 2010 in Netherland, the cardiac function of 51 patients having inclusion body myositis were assessed; systolic dysfunction was observed in 4 patients (8%), and 14 patients (27%) had diastolic dysfunction [12], supporting the fact that chronic inflammation due to other inflammatory diseases could impair myocardial function possibly due to similar mechanisms described for psoriatic patients.

The histologic hallmarks of psoriasis are epidermal hyperplasia and keratinocytes differentiation. Tumor necrosis factor alpha (TNF-α), dendritic cells, and T cells are believed to play roles in the pathogenesis of psoriasis; meanwhile, the detailed molecular pathogenesis of this disease is not thoroughly known [1]. Based on the gene mapping of HLA class 1, HLA-Cw6 allele is a predisposing factor [13].

Psoriasis and dilated cardiomyopathy have a similar genetic basis, and both are autoimmune and inflammatory diseases, which could explain the association between the two conditions; chronic production of pro-inflammatory cytokines in psoriasis may play a role in the pathogenesis of dilated cardiomyopathy [14]. Limited evidence has also indicated that children with psoriasis suffer from rhythm abnormalities and conduction disturbances. Although the pathogenesis of this disease is still not fully understood, inflammation is considered to be the most important mechanism for disease development and myocardial heterogeneity [15, 16]. Data also suggest that early-onset atherosclerosis and endothelial dysfunction are found in psoriatic patients without any known cardiovascular risk factors [17]. However, Alshami *et al*. demonstrated no correlation between psoriasis and non-ischemic dilated cardiomyopathy [18].

Of patients with dilated cardiomyopathy, myocarditis represents the most common identifiable etiology [19]. Myocarditis is a common cause of childhood heart failure. Although myocarditis and idiopathic dilated cardiomyopathy are considered distinct entities, myocarditis frequently presents with a phenotype of new-onset dilated cardiomyopathy [20]. The typical symptoms and signs at presentation in patients with acute myocarditis include chest pain, dyspnea, fatigue, palpitations, syncope, and cardiogenic shock. Acute myocarditis can also present as sudden cardiac death, accounting for approximately 10% of deaths from sudden cardiac death in young individuals (aged < 35 years) [19]; however, in children and adolescents (aged < 20 years), the related prevalence was reported 35% [21]. A previous study in sudden unexpected deaths in children and adolescents (1–20 years) found myocarditis to be the cause of cardiovascular death in 16/53 (30%) cases [22].

The diagnosis is challenging due to the heterogeneity of clinical presentations. A definite diagnosis requires endomyocardial biopsy, which is often still not part of routine practice [20]. Pathologic identification of an inflammatory cellular infiltrate is required for a definite diagnosis of myocarditis. While endomyocardial biopsy is typically well-tolerated, there are potential risks including the development of tricuspid valve regurgitation, arrhythmia, and cardiac perforation. These risks are likely magnified in small patients, and this is especially important considering children less than 1 year of age have the highest incidence of dilated cardiomyopathy [23].

The involvement of autoimmunity in inflammatory cardiomyopathy is well established. Inflammatory cardiomyopathy fulfils the Rose–Witebsky diagnostic criteria for organ-specific autoimmune disease [24]. Studies and registries of endomyocardial biopsy samples from patients with virus negative, chronic inflammatory cardiomyopathy suggest that the use of immunosuppressive therapy with prednisone and azathioprine can improve cardiac function. Alternative treatment regimens for patients with virus-negative or autoimmune inflammatory cardiomyopathy include steroid-based treatment combined with cyclosporine or mycophenolate mofetil, or immunoadsorption with subsequent intravenous immunoglobulin (IVIG) therapy (immunoadsorption–IVIG) [19]. Meanwhile, the present views on immunosuppressive therapy with steroids or immunomodulatory therapy with IVIG in children are still controversial [25].

There may be a correlation between the severity of psoriasis and comorbidities, suggesting that control of cutaneous disease may allow for better control of comorbidities. Systemic treatments for recalcitrant psoriasis are sometimes employed, but the majority are used off-label. These medications include methotrexate, cyclosporine, retinoids, and biological agents such as those targeting TNF-α and interleukin-12/23. These agents have been used successfully in other pediatric populations; however, they are still under investigation for children with psoriasis [26].

As the increased prevalence of cardiovascular diseases in patients having psoriasis results in decreased life expectancy among this population [27], the long-term management protocols must take into consideration this issue. Potential unmet needs in pediatric psoriasis include further delineation of diet and weight modification in the care and prevention of psoriasis; expansion of therapeutic trials and Unites States (US) Food and Drug Administration (FDA)–approved medications for children with psoriasis, especially severe variants such as extensive plaque and pustular disease; and development of guidelines for ongoing monitoring of children with psoriasis. The role of therapeutic interventions and weight management on long-term disease course remains to be shown in extended clinical trials [22].

Studies and registries of endomyocardial biopsy samples from patients with virus negative, chronic inflammatory cardiomyopathy suggest that the use of immunosuppressive therapy with prednisone and azathioprine can improve cardiac function [19], but the present views on immunosuppressive therapy with steroids in children are still controversial [25]. The successful treatment of cardiomyopathy in our patient using systemic glucocorticoids and azathioprine indicates that children could also benefit from systemic glucocorticoids similar to other age groups.

## Conclusions

In this study, treatment with anti-inflammatory drugs resulted in an improvement in heart failure, and EF was improved (65%). The results of this study add to evidence linking psoriasis with inflammatory dilated cardiomyopathy. In conclusion, these findings suggest that this patient population may be at an increased risk for cardiomyopathies. A standard 12-lead surface electrocardiogram, chest radiography, and if needed echocardiography could diagnose dilated cardiomyopathy in the context of psoriasis. However, further research is necessary to demonstrate the link between cardiomyopathies and pediatric psoriasis.

We recommend all clinicians take any sign or symptom suggesting heart failure in psoriatic patients seriously and perform appropriate investigations. We also call for large sample size, randomized controlled trials to evaluate the efficacy of treating inflammatory cardiomyopathy using systemic glucocorticoids and immunosuppressive agents including azathioprine, because of the limited numbers of associated studies on the topic.

## Data Availability

The datasets used during the current study are available from the corresponding author on reasonable request.
